# Maternal and Fetal Outcome of Obstetric Emergencies in a Tertiary Health Institution in South-Western Nigeria

**DOI:** 10.5402/2011/160932

**Published:** 2011-06-16

**Authors:** Lamina Mustafa Adelaja, Oladapo Olufemi Taiwo

**Affiliations:** ^1^Department of Obstetrics and Gynaecology, Olabisi Onabanjo University Teaching Hospital, Sagamu, Ogun State, Nigeria; ^2^Department of Obstetrics and Gynaecology, Gizan General Hospital, Gizan, Saudi Arabia

## Abstract

*Objective*. This study was carried out to determine the pattern of obstetric emergencies and its influence on maternal and perinatal outcome of obstetric emergencies at the Olabisi Onabanjo University Teaching Hospital (OOUTH), Sagamu, Nigeria. *Method*. A retrospective study of obstetric emergencies managed over a three-year period at Olabisi Onabanjo University Teaching Hospital (OOUTH), Sagamu, Nigeria was conducted. *Results*. There were 262 obstetric emergencies accounting for 18.5% of the 1420 total deliveries during the period. Unbooked patients formed the bulk of the cases (60.3%). The most common emergencies were prolonged/obstructed labour, postpartum haemorrhage, fetal distress, severe pregnancy-induced hypertension/eclampsia, and antepartum haemorrhage. Obstetric emergencies were responsible for 70.6% of the maternal mortality and 86% of the perinatal mortality within the period. *Conclusion*. Prevention/effective management of obstetric emergencies will help to reduce maternal and perinatal mortality in our environment. This can be achieved through the utilization of antenatal care services, making budget for pregnancies and childbirth at family level (pending the time every family participates in National Health Insurance Scheme), adequate funding of social welfare services to assist indigent patients, liberal blood donation, and regular training of doctors and nurses on this subject.

## 1. Introduction

An emergency can be defined as a situation of serious and often dangerous nature, developing suddenly and unexpectedly and demanding immediate attention in order to save life [1]. The maternal mortality ratio (MMR), expressed as maternal deaths per 100,000 live births over a given period, is a major measure of quality of obstetric care. According to World Health Organization (WHO) estimates, it varies up to 100-fold, from approximately 10 in developed countries to approximately 1,000 in least developed [2, 3]. Obstetric emergencies are the leading causes of maternal mortality worldwide and particularly in developing countries where literacy, poverty, lack of antenatal care, poor transport facilities and inadequate equipment/staffing combine to magnify the problem [4, 5]. Prevention where possible and prompt and effective treatment of obstetric emergencies will go a long way to reduce the magnitude of ever increasing maternal mortality which appears to have defied all proposed measures set to reduce it by WHO [6]. However, there are sparse data on the contribution of obstetric emergencies to maternal mortality in sub-Saharan Africa and developing countries like Nigeria where the maternal mortality ratios are very alarmingly high. This is one of the principal obstacles to appropriate distribution of resources targeted towards improving maternal healthcare. A study carried out by Nwobodo [7] in North-Western Nigeria showed that obstetric emergencies were responsible for 96.7% and 87% of the maternal and perinatal mortality, respectively. Although hospital-based studies have their limitations including referral bias, they are easy to perform in low-resource countries and can provide substantial and useful information [8, 9]. There have been several studies in South-Western Nigeria on maternal mortality [10–12] but none on obstetric emergencies in general and their influence on maternal and perinatal mortality. This study is therefore designed to explore this subject.

## 2. Subjects and Methods

### 2.1. Definition of Terms

In this study, an obstetric emergency is defined as an obstetric complication or situation of serious and often dangerous nature, developing suddenly and unexpectedly and demanding immediate attention in order to save life [1]. *Direct maternal deaths* are those resulting from complications of the pregnant state (pregnancy, labour, and puerperium), from interventions, from omissions, from incorrect treatment, or from a chain of events arising from any of the above while *indirect maternal deaths* are those due to a previously existing diseases or disease that develop during pregnancy, and not due to direct obstetric causes but which were aggravated by the physiological effects of pregnancy. An “*unbooked patient*” refers to a woman who did not utilize the antenatal care services of OOUTH, Sagamu, Nigeria. *Pregnancy-induced hypertension* is when a pregnant woman, who was normotensive before getting pregnant, develops high blood pressure without proteinuria during pregnancy. *Preeclampsia* is when a pregnant woman develops high blood pressure and proteinuria, usually after 20 weeks of gestation. *Eclampsia* is occurrence of fits in a preeclamptic patient.

### 2.2. Study Design, Data Collection, and Analysis

 This was a retrospective study in which the case records of all obstetric emergencies in OOUTH, Sagamu, Nigeria between January 2005 and December 2007 were obtained from the labour ward, antenatal ward, lying-in ward, and department of medical records of the hospital. Data extracted from the records include maternal age, parity, occupation, booking status, type of emergency and maternal/perinatal mortality. 

 The data was subsequently analysed, and where applicable, subjected to statistical analysis using SPSS 15.0 for Windows Evaluation Version. Level of significance was set at *P* < .05 and determined by *x*-squared analysis. This study was approved by The Scientific and Ethical Committee of OOUTH, Sagamu, Nigeria.

### 2.3. Hospital Setting

Ogun State is one of the 6 states in the southwestern region of Nigeria. The state has a land area of 2017 square kilometers and a population of 380,527. Ogun State has 20 local government areas of which Sagamu, with a population of 156,312 is one.

Olabisi Onabanjo University Teaching Hospital, Sagamu, Nigeria is the only tertiary hospital for referral from all clinics, maternity homes and hospitals in all the Local Government Areas in Remo and Ijebu areas of Ogun State and adjoining areas of Lagos State. The hospital is funded by the government of Ogun State in South-Western Nigeria. It provides emergency obstetric services to women referred from other centres in addition to providing antenatal care and delivery services for low and high risk pregnant women from Sagamu community and neighboring towns. Patients are expected to pay directly for their services (except few that participate in National Health Insurance Scheme) though in emergency situations, they are managed within the means of existing resources before funds are made available. The hospital provides blood transfusion services from limited stock, and relatives of patients are requested to donate or provide donors when blood transfusion is indicated.

## 3. Results

 During the three-year period, there were 262 obstetric emergencies out of 1420 total deliveries giving an incidence of 18.5%. One hundred and four (39.7%) were booked while 158 patients (60.3%) were unbooked for antenatal care and delivery. The maternal age ranged from 15 to 45 years with a mean of 30 ± 2 years. The parity ranged from 0 to 8 with a mean of 3 ± 1. The leading emergencies as shown in Table 1 were prolonged/obstructed labour, postpartum haemorrhage, fetal distress, severe pregnancy-induced hypertension/eclampsia and antepartum haemorrhage (placental praevia/abruptio placenta). Other important types of emergencies include puerperial sepsis, ruptured uterus and retained second twin. There were 17 maternal deaths within the period, and obstetric emergencies accounted for 12 deaths (70.6%). Eleven of the maternal deaths occurred among 158 unbooked patients (6.9%) while only one out of 104 booked patients (0.9%) died. The leading cause of maternal death was obstructed labour. The maternal death rate was statistically significantly higher in unbooked than booked patients (11 deaths in 158 unbooked patients versus 1 death in 104 booked patients; *P* < .05). Out of 50 perinatal deaths during the study period, obstetric emergencies were responsible for 43 (86%). The perinatal death rate was also significantly higher in unbooked than booked patients (43 deaths in 115 unbooked patients versus 7 deaths in 97 booked patients; *P* < .05). At the time of presentation, only 105 (40%) could afford about thirty-five dollars (five thousand naira) deposit for admission. Out of 64 patients that required immediate blood transfusion, only 20 (31.2%) had it within 2 hours. The causes of delay in the other group were failure of their relatives to donate blood or organize donors and lack of funds to pay for necessary investigations.

## 4. Discussion

This study has shown that obstetric emergencies were relatively common in this centre and unbooked patients constituted a substantial bulk of cases. Obstetric emergencies were responsible for most of the mortality within the period of study, and maternal death rate was higher among unbooked patients. This picture is similar in many tertiary institutions in Nigeria and other developing countries [5–20]. Therefore, if health facilities were able to adequately and effectively respond to the needs of these women, many of the fatal cases would probably have survived. With reference to maternal mortality, similar findings were reported by other authors in Nigeria [5–17, 19, 20]. Interestingly, obstructed/prolonged labor, eclampsia, obstetric hemorrhage, puerperal sepsis and ruptured uterus (which were the most common emergencies in this study) are among the leading causes of maternal and perinatal mortality in the country [10–17, 19, 20]. Similar findings were obtained by Nwobodo in North-Western Nigeria in 2006 [7].

This study reiterates the importance of proper antenatal care and delivery towards reducing maternal and perinatal mortality in this environment. Women unbooked for antenatal care and delivery were up to 22 times as likely to die in the hospital compared to booked patients [16, 20, 21]. The percentage of booked patients who died probably reflects the likely MMR if all women were to have adequate antenatal care and well-supervised delivery.

The leading causes of maternal and perinatal deaths in this study are not significantly different from those identified in the developing countries for several decades [10, 12–17, 19]. This implies that our pregnant women are still dying from preventable causes of maternal and perinatal deaths and unlike suggested by some authors [22], no special technology or research is required to tackle the problem in this part of the world.

Therefore it can be deduced that prevention (where possible) and effective management of obstetric emergencies will go a long way in reducing maternal and perinatal mortality in Nigeria. The strategies for achieving this objective will include the utilization of antenatal services, making budget for pregnancies and childbirth at family level (pending the time every family participates in National Health Insurance Policy), adequate funding of social welfare services to assist the indigent patients, the development of adequate blood banking system, liberal blood transfusion, and regular training of doctors and nurses on this subject.

**Limitations**

Inadequate documentation and the fact that some cases could have been missed because of the retrospective nature of this study made the compilation of statistics difficult in developing countries like ours. In addition, maternal deaths in the puerperium could have been underreported as postnatal clinic attendance in the hospital is generally poor and there is presently no measure to conduct home-based followup of parturients. This study only evaluates the effect of antenatal care (booked versus unbooked patients) on perinatal outcomes following an obstetric emergency among women admitted to hospital for labour and delivery. We do not know the outcome following an obstetric emergency among women excluded from hospital care for not being insured or able to pay for assistance.

##  Conflict of Interests

 The authors declare that they have no conflict interests.

## Figures and Tables

**Table 1:** Frequency of obstetric emergencies.

Type of emergency	*n* (%)
Prolonged labour	46 (17.6)
Obstructed labour	43 (16.4)
Postpartum haemorrhage	37 (14.1)
Fetal distress	28 (10.6)
Severe pregnancy-induced hypertension	23 (8.8)
Eclampsia	21 (8.0)
Antepartum haemorrhage	20 (7.6)
Puerperial sepsis	16 (6.1)
Ruptured uterus	12 (4.6)
Retained second twin	10 (3.8)
Severe anaemia in pregnancy	2 (0.8)
Cord prolapse	2 (0.8)
Cord presentation	1 (0.4)
Uterine inversion	1 (0.4)

Total	262 (100)

## References

[1] Campbell S, Lee C, Campbell S, Lee C (2000). Obstetric emergencies. *Obstetrics by Ten Teachers*.

[2] Hill K, AbouZahr C, Wardlaw T (2001). Estimates of maternal mortality for 1995. *Bulletin of the World Health Organization*.

[3] Buekens P (2001). Is estimating maternal mortality useful?. *Bulletin of the World Health Organization*.

[4] Drife J, Lueslay DM, Baker PN (2004). Maternal mortality. *Obstetrics and Gynaecology and Evidence-Based Text for MRCOG*.

[5] Chukwudebelu WO, Okonofua A, Odunsi K (2003). Preventing maternal mortality in developing countries. *Contemporary Obstetrics and Gynaecology for Developing Countries*.

[6] Haines A, Cassels A (2004). Can the millennium development goals be attained?. *British Medical Journal*.

[7] Nwobodo EL (2006). Obstetric emergencies as seen in a tertiary health institution in North-Western Nigeria: maternal and fetal outcome. *Nigerian Medical Practitioner*.

[8] Kampikaho A, Irwig LM (1991). Incidence and causes of maternal mortality in five Kampala hospitals, 1980–1986. *East African Medical Journal*.

[9] Geelhoed DW, Visser LE, Asare K, Leeuwen JHSV, Roosmalen JV (2003). Trends in maternal mortality: a 13-year hospital-based study in rural Ghana. *European Journal of Obstetrics Gynecology and Reproductive Biology*.

[10] Lamina MA, Adetoro OO, Odusoga OI, Fakoya TA, Adefuye PO (2001). A review of maternal mortality in Ogun state university teaching hospital, Sagamu, Nigeria. *African Journal of Medical and Pharmaceutical Sciences*.

[11] Akindele F, Roberts OA Maternal mortality at the university college, Ibadan: a ten-year review.

[12] Agboghoroma OC, Emuveyan EE (1997). Maternal mortality in Lagos, Nigeria: aten-year review (1986–1995). *Nigerian Quarterly Journal of Hospital Medicine*.

[13] Audu LR, Ekele BA (2002). A ten year review of maternal mortality in Sokoto, northern Nigeria. *West African Journal of Medicine*.

[14] Airede LR, Ekele BA (2003). Adolescent maternal mortality in Sokoto, Nigeria. *Journal of Obstetrics and Gynaecology*.

[15] Okoro JM, Umezulike AC, Onah HE Maternal mortality in the UNTH, Enugu “after Kenya”.

[16] Briggs ND (1998). Maternal death in the booked and unbooked patients; UPTH experience. *Tropical Journal of Obstetrics and Gynaecology*.

[17] Adetoro OO (1987). Maternal mortality—a twelve-year survey at the university of Ilorin teaching hospital (U.I.T.H.) Ilorin, Nigeria. *International Journal of Gynecology and Obstetrics*.

[18] Pokharel HP, Lama GJ, Banerjee B, Paudel LS, Pokharel PK (2007). Maternal and perinatal outcome among the booked and unbooked pregnancies from catchments area of BP Koirala institute of health sciences, Nepal. *Kathmandu University Medical Journal*.

[19] Adetoro OO, Okonofua F, Odunsi K (2003). Preventing perinatal mortality in developing countries. *Contemporary Obstetrics and Gynaecology for Developing Countries*.

[20] Okogbenin SA, Okonta PI, Eigbefoh J, Okusanya BO (2007). The demographic characteristics and health seeking behaviour of unbooked patients in Irrua specialist teaching hospital. *Nigerian Journal of Medicine*.

[21] Oladapo OT, Lamina MA, Fakoya TA (2006). Maternal deaths in Sagamu in the new millenium: a facility-based retrospective analysis. *BMC Pregnancy and Childbirth*.

[22] Tsu VD (2005). Appropriate technology to prevent maternal mortality: current research requirements. *International Journal of Obstetrics and Gynaecology*.

